# Nurse as a Facilitator to Professional Communication: A Qualitative Study

**DOI:** 10.5539/gjhs.v7n2p294

**Published:** 2014-11-16

**Authors:** Shahrzad Ghiyasvandian, Masoumeh Zakerimoghadam, Hamid Peyravi

**Affiliations:** 1 School of Nursing and Midwifery, Tehran University of Medical Sciences, Tehran, Iran; 2 School of Nursing and Midwifery, Iran University of Medical Sciences, Tehran, Iran

**Keywords:** professional communication, healthcare, nursing

## Abstract

Nurses need to establish communication with other healthcare professionals to facilitate the process of care. Healthcare professionals have complementary roles in providing care to patients. As the key members of the healthcare team, nurses also have an important role in establishing communication among other healthcare professionals. The final outcome of professional communication is effective care and improved patient outcomes. The aim of this study was to explore nurses’ role in establishing professional communications with other healthcare professionals. This was a descriptive qualitative study. The study was conducted by using the content analysis approach. A purposive sample of sixteen healthcare professionals was recruited from six teaching hospitals affiliated to Tehran University of Medical Sciences, Tehran, Iran. Study data were gathered by conducting personal face-to-face semi-structured interviews and were analyzed by using the qualitative content analysis approach. The three main themes of the study were ‘Nurse as the mediator of communication’, ‘Nurse as the executor of others’ duties, and ‘Nurse as a scapegoat’. Study findings can be used by nurses, managers, and health policy-makers to develop effective strategies for exactly determining and clarifying nurses and other healthcare professionals’ roles as well as for informing the public and other healthcare professionals about nurses’ roles and importance.

## 1. Introduction

Communication is the exchange of thoughts, ideas, or information through writing, speaking, body movements, and symbols and it includes all conscious or unconscious activities that individuals perform to affect other people (Chitty & Black, 2007). In other words, communication is the verbal and non-verbal exchange of information (Charlton et al., 2008). It is considered as the heart of all interactions (Chitty & Black, 2007).

Professional communication in clinical settings is healthcare professionals’ relationships with each other as well as their exchange of knowledge and skill for making wise clinical decisions about patient care (Kenaszchuk et al., 2010). According to Thompson and Stewart (2007), healthcare professionals clearly need to establish professional communication with each other (Thompson & Stewart, 2007). Nurses also cooperate with each other as well as with other healthcare professionals in designing and providing care to their clients (Apker et al., 2006).

Many factors can affect patient outcomes. One of these factors is professional communication (Manojlovich et al., 2009). Alongside with the increasing complexity of patient care and care delivery systems, healthcare professionals also need to communicate with each other for providing quality and safe care to patients (Tjia et al., 2009; Smith et al., 2012; Nadzam, 2009; Nair et al., 2012). Accordingly, providing quality care is greatly dependent on the healthcare professionals’ collective understanding of care plans which is in turn acquired through their effective communication with each other (Weber et al., 2009; Rothberg et al., 2012; Smith et al., 2012). The final outcome of clear and effective communication is quality and safe patient care (Chitty & Black, 2007; Apker et al., 2006; Thompson, & Stewart, 2007). Manojlovich et al. (2009) also found that effective communications among healthcare professionals, particularly between physicians and nurses, significantly affect the outcomes of intensive care (Manojlovich et al., 2009). In fact, effective professional communication is the fundamental prerequisite to quality nursing care (de Almeida Araujo et al., 2010).

On the other hand, ineffective professional communication can compromise the quality of care. Nazdam (2009) found that factors such as differences in individuals’ official positions, role and interpersonal conflicts, and power struggles can damage professional communications and hence, affect patient safety and care quality (Nadzam, 2009). Moreover, evidence shows that healthcare professionals’ ineffective professional communication increases the incidence of medical errors (Tjia et al., 2009; de Almeida Araujo et al., 2010; Thomas et al., 2009). Moreover, it can cause healthcare professionals to either miss vital nursing or medical procedures or repeat the unnecessary ones and hence, increase their occupational stress (Nadzam, 2009).

Nurses—as the key members of the healthcare team—need to establish effective professional communication with other healthcare professionals for providing quality care (Manojlovich & DeCicco, 2007). Mullan and Kothe (2010) noted that effective professional communication is the cornerstone of efficient nursing services (Mullan & Kothe, 2010). Compared with other healthcare professionals, nurses have more serious relationship with patients. Accordingly, they have a prominent coordinating role in providing care to patients and hence, their viewpoints and ideas about professional communication should be valued and taken into account (Vaismoradi et al., 2011). Moreover, due to the complexity of the problems and the difficulties that healthcare professionals experience during their daily practice, the process of professional communication is sometimes disrupted (Manojlovich & DeCicco, 2007). Hojat et al. (2001) reported that nurses’ professional communication with other healthcare professionals, particularly physicians, is strained and troubled (Hojat et al., 2001).

Nurses usually establish professional communications to facilitate the process of patient care and recovery. However, despite the great emphasis of nursing education on the process and the basics of professional communication, few comprehensive studies have been conducted so far on nurses’ communication skills (Apker et al., 2006). Given nurses’ different roles in establishing professional communications in different cultural and clinical settings as well as lack of comprehensive studies in the area of nurses’ communication skills, this study was conducted to explore nurses’ role in establishing professional communications with other healthcare professionals.

## 2. Methods

### 2.1 Design and Aim

This was a descriptive qualitative study which was conducted by using the content analysis approach. The aim of the study was to explore nurses’ role in establishing professional communications with other healthcare professionals.

### 2.2 Participants

Totally, sixteen healthcare professionals (ten nurses, two head nurses, two supervisors, and two resident physicians) participated in the study. Two participants had the experience of working in private hospitals and in head-nurse position. Study participants were recruited from the medical-surgical, emergency, and intensive care units of six teaching hospitals affiliated to Tehran University of Medical Sciences, Tehran, Iran. The sampling method was purposive. The inclusion criteria were having a one-year work experience in hospital and being willing to participate in the study. In order to include different viewpoints and experiences in the study, we strived to recruit a maximum variation sample in terms of variables such as gender, age, work experience, educational status, working shift, and working unit.

The data collection method was personal face-to-face semi-structured interview. Data were collected from March 2013 to April 2014. The length of the interviews was 40–80 minutes. According to the participants’ preferences, interviews were conducted in either the first author’s office or the head-nurse room located in participants’ workplace. Interviews were recorded and immediately transcribed verbatim. The main interview questions were, would you please explain about one of your workdays? What is your professional role in relationship with other healthcare professionals? Can you provide an example of your own professional communications? Moreover, probing questions were asked to further clarify the ambiguous points.

### 2.3 Data Analysis

The content analysis approach was used for data analysis (Graneheim & Lundman, 2004). Content analysis consists of advanced techniques for data processing. It is a systematic approach which aims at providing a novel insight and a better understanding about phenomena and helps identify their practical applications (Krippendorff, 2012). Interviews were transcribed verbatim. Each transcript was read for several times to achieve a basic understanding of it. Then, the transcript was divided into condensed meaning units. The condensed meaning units were abstracted and coded. Codes were compared and organized into sub-categories and categories according to their similarities and differences. Finally, we identified the latent content of the data and the main theme of the study by constantly comparing the generated sub-categories and categories and reflecting on them (Graneheim & Lundman, 2004).

### 2.4 Trustworthiness

Trustworthiness is among the most important issues in qualitative studies and facilitates the evaluation of researchers’ effects and actions (Holloway, 2005). Peer checking was used for establishing the credibility of the findings. Accordingly, we (i.e. the authors) coded and categorized the data independently. Then, the codes and the categories which had been generated by each author were compared with each other. In case of any disagreement, we discussed with each other to reach consensus. We also employed member-checking for maintaining credibility. A summary of the findings was provided to several participants and they were asked to confirm the similarity between the findings and their own experiences. Moreover, we used audit trailing for establishing the confirmability of the findings.

### 2.5 Ethical Considerations

The Ethics Committee of Tehran University of Medical Sciences approved this study (the approval code was 91D1303181). The objectives and the methods of the study were explained to the participants. We obtained informed consent from each participant. Moreover, we obtained their permission for recording the interviews. Participants were free to withdraw from the study without experiencing any disadvantage. They were assured that their information will be managed confidentially.

## 3. Finding

The means of participants’ age and work experience were 34.75 and 11.2 years, respectively. Table 1 shows the study participants’ characteristics. The three themes of the study were ‘Nurse as the mediator of communication’, ‘Nurse as the executor of others’ duties’, and ‘Nurse as a scapegoat’. The main themes of the study and the corresponding sub-themes are shown in Table 2 and are explained below.

**Table 1:** Participants’ characteristics

		Frequency(percentile)
**sex**	female	9(56.25%)
	male	7(43.75%)

**Age**	Mean	34.75
	SD	4.42

**Position**	nurse	10(62.5%)
	Head nurse	2(12.5%)
	Supervisor	2(12.5%)
	physician	2(12.5%)

**Marriage**	yes	8(50%)
	no	8(50%)

**Professional Background**	< 5 Years	3(18.75%)
	5-10 years	6(37.5%)
	> 10 years	7(43.75%)

**Table 2:** Themes and sub-themes of the study

**Nurse as the mediator of communication**	**Nurse at the core of communication**
	Nurse as the mediator of patient-managers communication
	Nurse as the mediator of patient-physician communication

**Nurse as the executor of others duties**	

**Nurse as a scapegoat**	Intra-professional unkindness toward nurses
	Inter-professional unkindness toward nurses
	Unfair treatment of nurses

### 3.1 Nurse as the Mediator of Communication

Compared with other healthcare professionals, Iranian nurses spend the greatest deal of time with patients and they are responsible for performing most care services. Our participating nurses were considered by physicians and other healthcare professionals as excellent sources of patient information. Moreover, they were easily accessible to patients. According to the study participants, the mediator role of nurses in communication reduced medical errors, enhanced patient safety, and improved the quality of care. This theme consisted of three sub-themes including nurse at the core of communication, nurse as the mediator of patient-managers communication, and nurse as the mediator of patient-physician communication. These sub-categories are explained below.

#### 3.1.1 Nurse at the Core of Communication

According to our participants, nurse is at the core of communication and plays a key role in facilitating professional communication. They viewed nurses as the link between patients and healthcare professionals.

*Nursing is at the core of healthcare providers’ communications. Nurse is a person to whom all other things are connected. In other words, a nurse should have communication with patients, physicians, and other healthcare providers*.

Our participants viewed nurses as professionals who are responsible for fulfilling all patients’ needs. Iranian nurses fulfill a wide range of responsibilities such as providing direct care to patients, coordinating patient care, relieving patients’ financial problems, and facilitating patients’ hospital discharge. For instance, when a patient is unable to pay care-related costs, nurses negotiate with social workers and managers of hospital to resolve patient’s financial problem. A participating supervisor said,

*Hospital nurse managers as well as social workers have an influential role in transferring patients from one ward to another. Some of our patients are homeless and have no family members. We often negotiate with hospital managers and ask them to manage such patients’ financial problems in such a way that they could be transferred from emergency ward to other wards of hospital*.

#### 3.1.2 Nurse as the Mediator of Patient-Managers Communication

Besides communicating with other healthcare professionals, our participating nurses also communicated with senior managers of their hospitals. For instance, lack of empty bed for patients who had been admitted to the emergency room resulted in the hospitalization of patients in other wards. Accordingly, nurses who were in charge for providing care to the newly-admitted patients were required to call for and find the attending physician who was resident in other wards. If they could not find the intended physician, they felt it necessary to call hospital manager(s). A participating supervisor highlighted,

*Sometimes, a patient is hospitalized in an irrelevant ward [a ward that is not directly relevant to patient’s underlying condition]. Accordingly, the attending physician can’t locate the patient and fails to visit him/her. In such occasions, we refer the problem to hospital managers and ask them to manage it*.

Accordingly, crowdedness of public hospitals, nurses’ heavy workload, and their involvement with non-nursing tasks compromised the quality of nursing care. A participating nurse noted,

*Our hospital is a public teaching health center and hence, it is very crowded. Despite suffering from a serious staff shortage, we need to spend our time on communicating with the management and performing others’ tasks instead of spending it on providing patient care*.

#### 3.1.3 Nurse as the Mediator of Patient-Physician Communication

Most of our participants pointed to nurses’ role in establishing and facilitating patient-physician communication. For instance, they needed to call physician to refer to hospital and visit patients who had been hospitalized during the night shift. Sometimes, physicians’ refusal to visit patients who had been hospitalized in irrelevant wards (such as a cancer patient in a cardiac care ward) caused our participating nurses great stress and deep trouble. One of the participating nurses mentioned,

*Patients who are hospitalized in irrelevant wards expand our workload. For these patients, we need to search for the attending physician. This process wastes our time and energy*.

A cardiac care nurse also noted,

*For instance, they had hospitalized a patient with brain tumor in our [cardiac care] ward. It was 03:00 AM. We spent a great deal of time on finding the attending physician. At the same time, we were worried about the deterioration of patient’s condition*.

The participating physicians also considered nurses as good facilitators to patient care. One of the physicians who participated in the study also referred to nurses as the bridge between patients and physicians by saying,

*I can’t work in the absence of nurses. Nurses are important mediators of patient-physician communication*.

It is noteworthy that despite the formidable barriers to nurses’ professional communications, they had accepted the role of being the mediator of patient-physician communication and strived for providing safe patient-centered care.

### 3.2 Nurse as the Executor of Others’ Duties

The second main theme of the study was nurse as the executor of others’ duties. Our participants felt deeply committed to providing timely and quality care to their patients as well as to fulfilling their needs. Accordingly, they occasionally chose to perform other healthcare providers’ tasks. In other words, they accepted multiple roles and shouldered heavier workload to fulfill nursing staff shortage out of their deep commitment to healthcare system and their patients. One of the participating head-nurses highlighted,

*I’m a head-nurse; however, my job description is different from the real job description of a head-nurse. For instance, one day one of my nurses was absent without permission while there were eleven patients in my ward. I felt compelled to perform all the tasks alone. I performed both my own and the absent nurse’s tasks*.

Moreover, our participating nurses were occasionally the executer of physicians’ tasks. According to them, some physicians just sign and stamp prescriptions, consultation request forms, or patients’ medical records without filling them completely and require nurses to fill them.

*We have the same problem with resident physicians. They partially fill a consultation request form without writing patient’s name or even bed number on it. If I don’t complete the form, patient’s surgery would be postponed*.

Informing patients about the courses and the prognoses of diseases is among physicians’ duties. However, our participating nurses were occasionally compelled to provide such information to patients and their families. Accordingly, they experienced a huger workload which finally resulted in their fatigue, burnout, and inability to fulfill their own duties. An internal medicine care nurse noted,

*A patient knows little, if any, about his/her disease. When doctors escape their responsibilities and delegate them to us, we are compelled to perform their duties and hence, experience heavier workload*.

Sometimes, nurses shouldered physicians’ responsibilities just for facilitating patients’ recovery. We asked one of the participants, ‘Why are physicians’ responsibilities placed on nurses?’ She answered,

*I prefer to manage patients’ affairs on my own. I perform physicians’ undone duties just for the sake of patients. I try to prevent patients from developing problems even if it causes me great difficulty*.

### 3.3 Nurse as a Scapegoat

The third main theme of the study was ‘nurse as a scapegoat’. According to our participants, nurses, in the process of professional communication, are treated unfairly. They are compelled to perform other healthcare professionals’ duties and hence, experience greater workload, occupational stress, and moral distress. Moreover, they do not receive adequate support from intra- or extra-professional authorities. In other words, they are treated with great unkindness. This theme covered three sub-themes including intra-professional unkindness toward nurses, inter-professional unkindness toward nurses, and unfair treatment of nurses.

#### 3.3.1 Intra-Professional Unkindness Toward Nurses

Our participating nurses have been occasionally made scapegoats for their junior and senior nursing colleagues and even supervisors’ failures and faults. An internal medicine care nurse mentioned,

*When I was working in the emergency ward, we received a letter from the Justice department concerning a patient who had died in the emergency ward. That patient had to be transferred to CCU for receiving intensive care; however, there was no empty bed in CCU. We asked the in-charge supervisor to find an empty CCU bed for him; however, we forgot to document the name of the supervisor in our written nursing report. The nursing management office of the hospital accused me and I have been made a scapegoat for that supervisor’s fault*.

#### 3.3.2 Inter-Professional Unkindness Toward Nurses

Our participants worked as the coordinator of care. Their most common inter-professional communication was with physicians. Sometimes, physicians showed our nurses great unkindness and caused them difficulties. A participant who worked in a surgical care unit said,

*Sometimes, we have a patient who is going to undergo a surgery in the following day. The attending physician requests a consultation with cardiologist. I spend a whole afternoon on calling the cardiologist and he refuses to visit the patient. Next day, I’m reprimanded for the undone cardiology consultation*.

According to the study participants, nurses sometimes cannot access the attending physician and hence, perform his/her duties to speed up the process of patient care and recovery and hence, suffer the negative consequences of such practice. We asked a participating nurse, ‘What were the consequences of performing physicians’ duties?’ He answered,

*I’ve seen nurses who had been reprimanded for performing physicians’ duties*.

Such negative consequences had caused our participants great occupational stress. Our participants reported that one of the sources of nurses’ occupational stress was physicians’ late patient visitation. In such cases, nurses are reprimanded by patients and their families. One of the participating nurses expressed,

Physicians rarely visit their patients timely and hence, we experience intense stress. A patient [whose doctor has been late] frequently asks us, ‘Why am I waiting here in vain?’

Our participants considered the physician-dominant culture of the Iranian healthcare system as the main reason for such problems. In this culture, physicians are viewed by public as saint and infallible people. Beside public, physicians themselves also have the same impression. Accordingly, nurses are considered as the scapegoats for physicians’ malpractice. Conversely, physicians are treated by patients and their families with utmost respect even if they fail to satisfy patients’ needs, expectations, and preferences.

*Before the arrival of attending physicians, patients constantly nag at us; but, when they see their physicians, they bow down to them and forget physicians’ unpunctuality. Unfortunately, all the burdens and pressures are on nurses because they are in the public’s eyes*.

The participating physicians also highlighted that the public has a positive image of physicians. One of our participants who was a physician noted,

*Of course, the public has a positive attitude towards physician and such an attitude is also transferred to us*.

Our participants’ experiences showed that nurses’ desperate need for establishing communication with others as well as their being easily accessible made them scapegoats. A participating nurse explained the reason behind nurses’ being scapegoats as follows,

*We are scapegoats because patients are in direct and close contact with us and hence, don’t appreciate us*.

Another participating nurse mentioned,

*Patients are mostly in relation with us. Accordingly, they consider us as being responsible for all their affairs*.

#### 3.3.3 Unfair Treatment of Nurses

According to the study participants, patients and their families attribute all the shortcomings of hospital system to nurses. Such unfair prejudice about nurses makes them scapegoats for the shortcomings that are irrelevant to them. The supervisor of an emergency ward said,

*Many patients have no insurance and hence, their discharge is postponed. In such cases, supervisors or nurses are accused. Patients ignore all other people—from physicians to social workers and managers—who have contributed to their prolonged hospital stay and just accuse me*.

Our participants believed that because of such unfair treatment, nurses usually suffer oppression and are accused for any faults that occur in hospitals and clinical settings.

*Nurses have been oppressed. All responsibilities are on their shoulders. They should work harder to compensate for staff shortage. However, people, unfortunately, attribute faults and weaknesses to them*.

Unfair treatment of nurses was not only limited to poor impressions and inaccurate judgments but it was also manifested in behaviors. According to our participants, the reason for such unfair treatments was the poor public image of nursing.

*Nurses facilitate the process of care delivery and fulfill all the necessary requirements. However, when patients achieve recovery, they only appreciate physicians and neglect nurses*.

Another participating nurse referred to people’s great confidence in physicians as the main reason for attributing all faults and weaknesses to nurses and expressed,

*As patients have confidence in physicians, they always question our abilities and competence even if we are right*.

Generally, our participating nurses, in the process of professional communication, highly valued and strived for on-time delivery of quality care. Accordingly, they chose to shoulder a heavier workload and perform other healthcare professionals’ duties for facilitating the process of on-time care delivery. Nonetheless, they were subjected to both intra- and inter-professional unkindness, treated unfairly, and accused wrongly.

## 4. Discussion

This study aimed at exploring nurses’ role in establishing professional communications with other healthcare professionals. Study findings revealed that nurses act as the facilitators to professional communications. The first theme of the study revealed that nurses are the mediators of professional communications. In other words, they are at the core of professional communication and hence, have an important role in establishing and facilitating it. Communication in nursing is so much important that it is considered as a primary component in care and a central task of nurses (Naish, 1995; Wallace, 2001). Nurses participate in and manage the designing and implementing systems that support effective team work (Pfaff et al., 2014). Accordingly, they need to establish communications with intra- and extra-professional managers. Wallace (2001) also noted that nurses are the key members of the healthcare team and play a pivotal role in helping the team achieve its aims (Wallace, 2001).

Study findings also showed that compared with other healthcare professionals, nurses spend the greatest deal of time with patients and hence, they are at the forefront of communication and are the mediator of patient-physicians relationship. Niekerk and Martin (2002) also noted that nurses, as the link between patients and physicians, can greatly influence the processes of diagnosis, treatment, and recovery (Van Niekerk & Martin, 2002). Accordingly, effective nurse-physician professional communication positively affects care quality, patient outcomes, and patient satisfaction (Tjia et al., 2009; Smith et al., 2012; Nair et al., 2012; Nadzam, 2009).

We also found that nurses are the executors of others’—including other nurses, physicians, and laboratory staffs’—duties. They chose to perform others’ duties out of conscientiousness in order to accelerate the process of patient care and recovery. Another reason was the inaccessibility of physicians to patients. Tjia et al. (2009) also reported similar findings (Tjia et al., 2009). They found that the major barriers to professional communication were physicians’ hurriedness and patients’ restricted access to them. Moreover, our participants were compelled to perform others’ duties because of role ambiguities. DeValk and Oostrom (2007) noted that role ambiguity can increase healthcare professionals’ stress. They also highlighted that nurses’ conscientiousness, their concern over the possibility of unsafe practice, and other healthcare professionals’ idealistic approach to care increase nurses’ occupational stress and workload (De Valk & Oostrom, 2007). Our participants also reported to have stress, anxiety, and increased workload. According to James et al. (2007), unequal role expectation is a major barrier to good professional communications, particularly between nurses and physicians (Casanova et al., 2007). Unclear role expectations and imprecise job specifications significantly contribute to nurses’ job dissatisfaction and turnover (Tunc & Kutanis, 2009). Conversely, precise role determination and clarification, strong professional identity, and mutual inter-professional respect improve professional communications (Pullon, 2008).

The third theme of the study was nurse as a scapegoat. Patients expect nurses to fulfill all their needs because they are easily accessible. Accordingly, nurses are made scapegoats for their nursing and non-nursing colleagues’ faults and malpractices. Rothberg et al. (2012) found that nurses are at the forefront of communication and conversely, physicians spend little amount of time on communicating with patients and other healthcare professionals (Rothberg et al., 2012). Study findings also revealed that in the process of professional communication, nurses are occasionally treated with intra- and inter-professional unkindness. According to Robinson et al. (2010), mutual support and respect improve effective professional communications (Robinson et al., 2010). On the other hand, lack of formal and informal supports in stressful situations is a major barrier to professional communication (Pfaff et al., 2014). According to James et al. (2007), although nurses greatly value partnered communication as a key component of successful patient care, they usually do not employ it in their daily clinical practice (Casanova et al., 2007). Moreover, we found that other healthcare professionals as well as patients and their families treat nurses unfairly. Many people believe that only physicians are aware of patients’ health status and hence, they only adhere to physicians’ orders. Such attitude towards physicians makes them feel that they maintain a patriarchal domination over care delivery and have control over all care-related affairs (Baiyekusi, 2010; Mannahan, 2010). Accordingly, they trivialize nurses’ work.

## 5. Conclusion

In this study, we strived to explore nurses’ role in establishing professional communications with other healthcare professionals. Study findings indicate that nurses are facilitators to professional communication. Given the pivotal role of nurses in healthcare team, they mediate healthcare professionals’ communication with each other and with patients. Nurses’ communication-related roles increase their workload and compel them to occasionally perform other healthcare professionals’ duties. Such practice increases their professional accountability and occupational stress. Consequently, paying closer attention to nurses’ role in professional communication for enhancing the quality of care and nurses’ job satisfaction is crucial. Study findings can be used by nurses, managers, and health policy-makers to develop effective strategies for identifying and clarifying nurses and other healthcare professionals’ roles as well as for informing the public and other healthcare professionals about nurses’ roles and importance.

## Study Limitations and Recommendations

This study explored only the experiences and the viewpoints of healthcare professionals who were affiliated to teaching hospitals. Accordingly, conducting studies for exploring experiences and viewpoints of healthcare professionals who are working in different public and private healthcare settings is needed. Moreover, exploring experiences of nurses who are working in different care settings—including operating rooms, emergency wards, and intensive care units— is recommended. Comparing nurses’ role expectations with their job specifications by using quantitative methods would be another area of investigation. Also exploring experiences of more physicians is recommended.
